# Cish actively silences tcr signaling in CD8+ T cells to maintain tumor tolerance

**DOI:** 10.1186/2051-1426-3-S2-P39

**Published:** 2015-11-04

**Authors:** Douglas Palmer, Geoff Guittard, Zulmarie Franco, Shashank Patel, Christopher A Klebanoff, Madhusudhanan Sukumar, Robert L Eil, David Clever, Lakshmi Balagopalan, Rahul Roychoudhuri, Larry Samelson, Nicholas Restifo

**Affiliations:** 1 NIH/NCI - Surgery Branch, Bethesda, MD, USA; 2 NCI, Bethesda, MD, USA; 3 Center for Cancer Research, NCI/NIH, Bethesda, MD, USA

## Background

Improving the functional avidity of effector T cells is critical in overcoming inhibitory factors within the tumor microenvironment and eliciting tumor regression.

## Methods

We have found that Cish, a member of the Suppressor of Cytokine Signaling (SOCS) family, is induced by TCR stimulation in CD8^+^ T cells and inhibits their functional avidity against tumor.

## Results

Genetic deletion of *Cish* in CD8^+^ T cells enhances their expansion, functional avidity and cytokine polyfunctionality, resulting in pronounced and durable regression of established tumors. Although Cish is commonly thought to block STAT5 activation, we found that the primary molecular basis of Cish suppression is through inhibition of TCR signaling. Cish physically interacts with the TCR intermediate PLCg1, targeting it for proteasomal degradation following TCR stimulation. Furthermore we extend these findings to patients PBL retrovirally transduced with tumor-specific TCRs and shorthairpin microRNAs targeting *CISH*.

## Conclusions

These findings establish a novel targetable interaction that regulates the functional avidity of tumor-specific CD8^+^ T cells and can be manipulated to improve adoptive cancer immunotherapy.

